# Prevalence and outcome of bloodstream infections due to third-generation cephalosporin-resistant Enterobacteriaceae in sub-Saharan Africa: a systematic review

**DOI:** 10.1093/jac/dkz464

**Published:** 2019-11-19

**Authors:** Rebecca Lester, Patrick Musicha, Nadja van Ginneken, Angela Dramowski, Davidson H Hamer, Paul Garner, Nicholas A Feasey

**Affiliations:** 1 Liverpool School of Tropical Medicine, Liverpool, UK; 2 Malawi-Liverpool-Wellcome Trust Clinical Research Programme, Blantyre, Malawi; 3 Centre for Tropical Medicine and Global Health, Nuffield Department of Medicine, University of Oxford, Oxford, UK; 4 Mahidol-Oxford Tropical Medicine Research Unit, Mahidol University, Bangkok, Thailand; 5 Department of Health Services Research, University of Liverpool, Liverpool, UK; 6 Department of Paediatrics and Child Health, Stellenbosch University, Cape Town, South Africa; 7 Departments of Global Health and Medicine, Boston University Schools of Public Health and Medicine, Boston, MA, USA

## Abstract

**Background:**

The prevalence of bacterial bloodstream infections (BSIs) in sub-Saharan Africa (sSA) is high and antimicrobial resistance is likely to increase mortality from these infections. Third-generation cephalosporin-resistant (3GC-R) Enterobacteriaceae are of particular concern, given the widespread reliance on ceftriaxone for management of sepsis in Africa.

**Objectives:**

Reviewing studies from sSA, we aimed to describe the prevalence of 3GC resistance in *Escherichia coli*, *Klebsiella* and *Salmonella* BSIs and the in-hospital mortality from 3GC-R BSIs.

**Methods:**

We systematically reviewed studies reporting 3GC susceptibility testing of *E. coli, Klebsiella* and *Salmonella* BSI. We searched PubMed and Scopus from January 1990 to September 2019 for primary data reporting 3GC susceptibility testing of Enterobacteriaceae associated with BSI in sSA and studies reporting mortality from 3GC-R BSI. 3GC-R was defined as phenotypic resistance to ceftriaxone, cefotaxime or ceftazidime. Outcomes were reported as median prevalence of 3GC resistance for each pathogen.

**Results:**

We identified 40 articles, including 7 reporting mortality. Median prevalence of 3GC resistance in *E. coli* was 18.4% (IQR 10.5 to 35.2) from 20 studies and in *Klebsiella* spp. was 54.4% (IQR 24.3 to 81.2) from 28 studies. Amongst non-typhoidal salmonellae, 3GC resistance was 1.9% (IQR 0 to 6.1) from 12 studies. A pooled mortality estimate was prohibited by heterogeneity.

**Conclusions:**

Levels of 3GC resistance amongst bloodstream Enterobacteriaceae in sSA are high, yet the mortality burden is unknown. The lack of clinical outcome data from drug-resistant infections in Africa represents a major knowledge gap and future work must link laboratory surveillance to clinical data.

## Introduction

The emergence and spread of antimicrobial resistance (AMR) in bacteria is recognized as a global public health problem.^1^ Drug-resistant infections (DRIs) caused by AMR bacteria threaten human health worldwide, with the greatest mortality burden expected to occur in low- and middle-income countries.^2^ In settings where antibiotics and advanced diagnostics are available and affordable, DRIs can be treated with tailored regimens using second- or third-line antibiotics; however, these agents cost more and increase healthcare expenditure.^3^ In sub-Saharan Africa (sSA), where bacterial bloodstream infection (BSI) is a major cause of morbidity and mortality,^4^ diagnostic facilities are scarce and antibiotics such as carbapenems and semi-synthetic aminoglycosides (e.g. amikacin) are either unavailable or prohibitively expensive, the morbidity and mortality from DRIs is predicted to be high.^2^^,^^5^

In many sSA hospitals, limited nursing capacity favours the use of broad-spectrum antimicrobials with a once-daily dosing regimen and this has led to the widespread adoption of the third-generation cephalosporin (3GC) ceftriaxone for the empirical management of hospitalized patients with suspected sepsis.^6^ ESBL-producing Enterobacteriaceae, which are resistant to penicillins and 3GCs, represent a threat to the treatment of BSI in this setting and have been identified as priority pathogens on which all national AMR programmes should focus their surveillance and reporting.^2^^,^^7^

Comprehensive AMR surveillance in sSA is limited by lack of quality-assured diagnostic microbiology laboratories, but knowledge of the prevalence and spatiotemporal trends of 3GC-resistant (3GC-R) Enterobacteriaceae is critical to inform national and international antibiotic prescribing guidelines. Additionally, securing access to effective second- and third-line antibiotics in Africa will not only require an understanding of the prevalence of 3GC resistance, but also of the burden and impact of these pathogens on patients and healthcare systems.^8^ We have therefore systematically reviewed published reports of 3GC susceptibility amongst key Enterobacteriaceae in sSA, including surveillance data and clinical cohorts. Robust clinical outcome data are needed to support the estimates and assumptions that the greatest global burden associated with AMR will occur in sSA^5^ and we have therefore also reviewed studies that describe mortality from 3GC-R BSI. The aim of this systematic review was to determine the prevalence of 3GC resistance amongst *Escherichia coli*, *Klebsiella* spp. and *Salmonella* BSI in sSA and to provide an estimate of the associated mortality burden from these infections.

## Methods

### Search strategy and selection criteria

We systematically reviewed articles published between 1 January 1990 and 31 August 2019, according to a pre-specified protocol, prepared in February 2017 (Table S1, available as Supplementary data at *JAC* Online) with no language restrictions, following PRISMA guidelines (Table S2). We searched PubMed and Scopus according to a predefined strategy with search terms relating to BSI and susceptibility testing (Table S3). A search string that included all sSA countries as defined by the UN list of 54 African sovereign states returned more articles than a string using ‘Africa’ alone. References cited in selected articles were reviewed for additional articles and authors were contacted to obtain original data, where percentages but not absolute numbers of resistant organisms were provided.

Studies were included if they tested *E. coli, Klebsiella* spp. or *Salmonella* spp. for 3GC resistance. Methods of confirmatory ESBL testing, such as double-disc synergy or PCR, were extracted from articles if they were reported, but we did not exclude studies that did not confirm ESBL status. We included surveillance data in addition to studies reporting clinical cohorts, but excluded case reports, case series, expert opinions and reviews.

### Data extraction

Two authors (R.L. and P.M.) independently searched the literature and screened the abstracts of all retrieved records. The full text of remaining English articles was reviewed by one author (R.L.) and of French language articles by another (N.V.G.). Articles in other languages were not found in the search. Disputes about article inclusion were resolved through discussion, with recourse to a third reviewer (N.A.F.) if required. Predefined variables were extracted from each article (Table 1). Variables included study design and setting, clinical data such as age and HIV prevalence of clinical cohorts, and information on laboratory methods including antimicrobial susceptibility testing (AST) method and guideline, and method of ESBL confirmation. Mortality data were extracted as they were reported in the articles, as case-fatality rates, ORs or relative risks (RRs).

**Table 1:** Characteristics of included studies

First author	Country, year of publication	Years of data collection	Study type	Healthcare setting	Age category	HIV, *n* (%)	Blood culture method, organism identification	AST method, AST breakpoint guideline	ESBL confirmatory test	External lab QC	Blood culture positivity in study population, *n* (%)	Prevalence of 3GC resistance, *n* (%)	Other findings
Acquah^41^	Ghana	2011–12	Retrospective analysis of positive blood cultures	Urban referral hospital	Paediatric	NR	Manual	Disc diffusion	NR	Yes	86/331 (26.0)	*Klebsiella* spp. 1/12 (8.3)	
	2013						Manual	CLSI					
Apondi^42^	Kenya	2002–13	Retrospective analysis of *Klebsiella* isolates	Urban referral hospital	All ages	NR	Automated	Disc diffusion	NR	Yes	NR	*Klebsiella* spp. 68/78 (87.2)	
	2016						NR	CLSI					
Bejon^43^	Kenya	1994–2001	Retrospective analysis of Gram-negative bacilli	Rural district hospital	Paediatric	NR	Manual (<1998) then automated	Etest	NR	NR	NR	*E. coli* 0/141	
	2005											*Klebsiella* spp. 4/63 (6.0)	
							NR						
												NTS 0/296	
Blomberg^17^	Tanzania 2007	2001–02	Prospective cohort of children with suspected systemic infection	Urban referral hospital	Paediatric (0–7 years)	(16.8)	Automated Manual	Disc diffusion and Etest	Etest, PCR	NR	255/1828 (13.9)	*E. coli* 9/37 (24.3) *Klebsiella* spp. 9/52 (17.0) NTS 1/39 (2.6)	Significantly higher 3GC resistance in HAI *E. coli* than CAI
								CLSI					


Breurec^44^	Senegal	2007–08	Prospective cohort of neonates with suspected systemic infection	Urban referral hospitals (three sites)	Paediatric (neonates)	NR	Manual	Disc diffusion	Double-disc synergy	NR	77/226 (34.0)	*Klebsiella* spp. 33/39 (84.6)	Distinguish EOS from LOS but difference in 3GC resistance NR
	2016						Manual	FSM					
Brink^45^	South Africa	2006	Prospective review of bacterial isolates	Private urban hospitals (12 sites)	All ages	NR	NR	Mixture of disc diffusion and automated (VITEK 2)	Mixture of VITEK 2 and double-disc synergy	Yes	NR	*E. coli* 47/471 (10.0)	
	2007											*Klebsiella* spp. 293/636 (46.0)	
								CLSI					
Buys^21^	South Africa	2006–11	Retrospective review of *K. pneumoniae* isolates	Urban referral hospital	Paediatric	82/410 (20.0)	Automated	Mixture of VITEK 2, disc diffusion and Etest CLSI	Mixture of VITEK and double-disc synergy	NR	NR	*Klebsiella* spp. 339/410 (83.0)	Higher 3GC resistance in HAI than HCAI or CAI
	2016						Automated (VITEK 2)						
													Reports trends but no definite pattern over time
Crichton^46^	South Africa	2012–15	Cross-sectional review of BSI	Urban referral hospital	Paediatric	18/141 (12.8)	Automated	Mixture VITEK/disc diffusion	NR	Yes	938/7427 (12.6)	*E. coli* 8/36 (22)	Possibly higher 3GC resistance in CAI but no statistical analysis
	2018						Automated (VITEK 2)						
								CLSI					
Dramowski^47^	South Africa	2009–13	Retrospective cohort of HA neonatal BSI	Urban referral hospital	Paediatric (neonates)	NR	Automated	VITEK 2	NR	Yes	717/6251 (11.5)	*E. coli* 7/58 (12.1)	All HAI
							Automated (VITEK 2)	CLSI					
												*Klebsiella* spp. 172/235 (73.2)	
	2015a												
Dramowski^10^	South Africa	2008–13	Retrospective review of paediatric BSI	Urban referral	Paediatric (excluding neonates)	(13.4)	Automated	VITEK 2	NR	Yes	935/17 001 (5.5)	*E. coli* 12/97 (12.4)	No significant difference in 3GC resistance between HAI and CAI; no increase in 3GC resistance over study period
							Automated (VITEK 2)	CLSI					
	2015b											*Klebsiella* spp. 122/158 (77.2)	
Eibach^20^	Ghana	2007–09	Prospective cohort of patients with fever/history of fever or suspected neonatal sepsis	Rural district hospital	All	NR	Automated	VITEK 2	Double-disc synergy and PCR	Yes	NR	*E. coli* 5/50 (10)	Possible lower 3GC resistance in CAI, but no statistical analysis
	2016	2010–12					Mixed (API with MALDI-TOF confirmation)	EUCAST				*Klebsiella* spp. 34/41 (82.9)	
												NTS 0/215	
Jaspan^48^	South Africa 2008	2002–06	Retrospective cohort of HIV-infected children	Urban referral	Paediatric (3 months–9 years)	(100)	NR Manual	Disc diffusion ± Etest	NR	NR	NR	*Klebsiella* spp. 11/11 (100)	All *Klebsiella* were HAI
								CLSI					
Kalonji^13^	DRC	2011–14	Multisite prospective surveillance of *Salmonella* BSI	Mixed urban referral and private	Paediatric (excluding neonates)	NR	Manual	Disc diffusion	Double disc synergy and PCR	Yes	2353/14 110 (16.7)	NTS 49/776 (6.3)	
	2015						Manual	CLSI					
												*S.* Typhi 0/164	
Kariuki^49^	Kenya 2006	2002–05	Prospective cohort of children with NTS in blood/CSF or stool	Urban referral and private hospital	Paediatric (4 weeks to 84 months)	NR	Manual Manual	Disc diffusion and Etest	Double-disc synergy	Yes	NA	NTS 0/198	
								CLSI					
Kariuki^49^^,^^50^	Kenya	1994–2005	Cross-sectional review of NTS isolates over 12 years	Rural district hospital	Children (0–13 years)	NR	NR	Disc diffusion	Double-disc synergy	Yes	NA	NTS 0/336	Trends reported, no change over time
	2006						Manual	CLSI					
Ko^16^	South Africa	1996–97	Prospective cohort of patients with CA *K. pneumoniae*	Urban multisite	Adults >16 years	7/40 (18)	NR	NR	Broth dilution or double-disc synergy	NR	NA	*K. pneumoniae* 3/40 (7.5)	CAI only
							Automated (VITEK 2)	NR					
	2002												
Kohli^51^	Kenya	2003–08	Retrospective analysis of positive blood cultures	Urban referral	All	123/1092 (11.3)	Automated	Disc diffusion	NR	Yes	1092/18 750 (5.8)	*E. coli* 10/69 (14.5)	
	2010						Manual	CLSI					
												*Klebsiella* spp. 5/38 (13.1)	
												NTS 0/143	
Labi^52^	Ghana	2010–13	Retrospective review of *Salmonella* blood culture isolates	Urban referral	All	NR	Automated	Disc diffusion	NR	Yes	2768/23 708 (11.7)	NTS 12/198 (6.1)	
	2014						Manual	CLSI					
Lochan^53^	South Africa 2017	2011–13	Retrospective cohort of children with culture-confirmed BSI	Urban referral	Paediatric	17/524 (13.4)	Automated Automated (VITEK 2)	VITEK 2, disc diffusion and Etest CLSI	VITEK 2 or double-disc synergy	NR	958/16 951 (5.7)	*E. coli* 31/92 (33.7) *Klebsiella* spp. 68/88	No obvious difference in 3GC resistance between CAI, HAI and HCAI but no statistical analysis
		

Lunguya^54^	DRC	2007–11	Prospective cohort of invasive NTS	Mixed multisite—full details NR	All	NR	Manual	VITEK 2	VITEK and double-disc synergy	Yes	989/9364 (10.3)	NTS 3/233 (1.3)	
	2013						Manual with VITEK 2 confirmation	CLSI					
Mahende^14^	Tanzania	2013	Prospective cohort of children with fever or history of fever	Rural district hospital	Paediatric (2–59 months)	NR	Automated	Disc diffusion	NR	Yes	26/808 (3.2)	*S*. Typhi 1/17 (5.9)	
	2015						Manual	CLSI					
Maltha^15^	Burkina Faso	2012–13	Prospective cohort of children with fever or signs of severe illness	Rural district hospital and health centre	Paediatric <15 years	8/711 (1.1)	Automated	Disc diffusion	Double-disc synergy	NR	63/711 (8.9)	NTS 1/21 (4.8)	
							Manual	CLSI				*S*. Typhi 0/12	
	2014												
Marando^22^	Tanzania	2016	Prospective cohort of neonates with suspected sepsis	Rural district hospital	Neonates	NR	Manual	Disc diffusion	Double-disc synergy	NR	60/304 (19.7)	*Klebsiella* spp. 21/26 (80.8)	
	2018						Manual	CLSI					
Mengo^12^	Kenya	2004–06	Cross sectional study of *S.* Typhi isolates	Urban referral and private	All	NR	NR	Disc diffusion	NR	NR	NA	*S*. Typhi 6/100 (6.0)	
	2010							CLSI					
Mhada^55^	Tanzania	2009–19	Prospective cohort of neonates with suspected sepsis	Urban referral hospital	Neonates	NR	Manual	Disc diffusion	NR	NR	5/330 (1.5)	*E. coli* 2/14 (14.3)	Differentiates LOS and EOS but not by AMR patterns
	2012						Manual	CLSI					
												*Klebsiella* spp. 4/22 (18.2)	
Morkel^56^	South Africa	2008	Retrospective cohort of positive blood cultures on NICU	Urban referral hospital	Paediatric (neonates)	HIV exposed 9/54 (16.6)	NR	NR	NR	NR	58/503 (11.5)	*Klebsiella* spp. 10/17 (58.8)	
	2014												
Mshana^57^	Tanzania	NR	Cross-sectional review of Gram-negative isolates from blood/urine/swabs	Urban referral hospital	NR	NR	NR	Disc	Double disc synergy	Yes	NR	*Klebsiella* spp. 29/31 (93.5)	
	2009							CLSI					
Musicha^6^	Malawi	1998–2016	Retrospective isolate surveillance from patients admitted with suspicion of sepsis	Urban referral hospital	All	NR	Automated	Disc	Double disc synergy	Yes	29 183/194 539^58^	*E. coli* 140/1311 (10.7)	Trends show increase in 3GC resistance over time
	2017						Manual, confirmed with WGS	CLSI					
												*Klebsiella* spp. 260/542 (48.0)	
Ndir^11^	Senegal	2012–13	Case–control of patients with Enterobacteriaceae in blood	Urban referral	Paediatric	NR	NR	Disc	Double disc		173/1800 (9.6)	*E. coli* 7/12 (58.3)	HAI only
	2016						Manual	FSM					
												*Klebsiella* spp. 33/40 (82.5)	
Obeng- Nkrumah^59^	Ghana	2008	Prospective cohort of patients with Enterobacteriaceae in blood culture	Urban referral	All ages	NR	Automated	Disc diffusion	Double disc	NR	NR	*E. coli* 5/17 (29.4)	
	2013						Manual	CLSI					
												*Klebsiella* spp. 13/26 (50.0)	
			Culture criteria NR										
Obeng- Nkrumah^60^	Ghana	2010–13	Retrospective analysis of children with BSI	Urban referral	Paediatric (excluding neonates)	NR	Automated	Disc diffusion	NR	NR	1451/15 683 (9.3)	*E. coli* 63/112 (56.2)	
	2016						Manual	CLSI					
												*Klebsiella* spp. 40/68 (58.8)	
Ogunlesi^61^	Nigeria	2006–08	Mixed prospective/retrospective cohort of neonates with presumed or probable sepsis	Urban referral	Neonates	NR	Broth	Disc diffusion	NR	Yes	174/1050 (16.6)	*E. coli* 6/16 (37.5)	
	2011							CLSI					
												*Klebsiella* spp. 12/33 (36.4)	
Oneko^62^	Kenya	2009–13	Prospective cohort of children with invasive NTS (nested cohort in RTS,S trial)	Rural district	Paediatric (6–12 weeks and 5–17 months)	131/1696 (7.7)	Automated	Disc diffusion and broth microdilution	NR	Yes	134/1692 (7.9)	NTS 17/102 (16.7)	
	2015						Manual						
								CLSI					
Onken^19^	Tanzania (Zanzibar)	2012–13	Prospective cohort of patients with suspected systemic infection	Urban referral	All ages	NR	Manual, confirmed with automated	Mixed disc diffusion, confirmed with VITEK 2	ESBL Etest and PCR	Yes	66/470 (14.0)	*E. coli* 1/10 (10)	
												*Klebsiella* spp. 5/11 (45.5)	
	2015												
							Manual	EUCAST					
Paterson^63^	South Africa	1996–97	Prospective cohort of patients with *K. pneumoniae* BSI	Urban multisite	Adults >16 years of age	NR	Mixed	NR	Broth dilution	NR	NR	*Klebsiella* spp. 28/76 (37.0)	HAI only
													Reports mortality data for 3GC resistance but not split by country
	2004												
			Part of multi-country surveillance										
Perovic^64^	South Africa	2010–12	Multisite prospective surveillance of *K. pneumoniae* isolates	Academic urban centres (multisite)	All	NR	NR	MicroScan	14% confirmed with PCR from each region	NR	NR	*Klebsiella* spp. 1895/2774 (68.3)	Reports trends with increase over 3 years
							Automated (VITEK 2)	CLSI/EUCAST and/or MicroScan guidelines					
	2014												
Preziosi^65^	Mozambique	2011–12	Prospective cohort of adults with fever	Urban referral hospital	Adults ≥18 years	652/841 (77.5)	Automated	Disc diffusion	Double-disc synergy	NR	63/841 (7.5)	*E. coli* 1/14 (7.1)	
							Manual	CLSI					
	2015	2013–14											
												NTS 4/10 (40.0)	
Sangare^66^	Mali	2014	Prospective cohort, patients with suspected systemic infection, referred from other health centres	Urban referral hospital	All	NR	Automated	Disc diffusion	Double disc	Yes	NR	*E. coli* 8/34 (23.5)	Referral patients only but not defined as HAI
	2016						Manual with VITEK /MALDI-TOF confirmation	EUCAST					
												*Klebsiella* 10/34 (29.4)	
Seboxa^18^	Ethiopia	2012–13	Prospective cohort of adults with clinically suspected sepsis and retrospective study of blood cultures positive for Gram-negative bacilli	Urban referral	All	123/399 (30.1)	Automated (manual for retrospective cohort)	Disc diffusion	NR	NR	38/299 (12.7)	*E. coli* 8/16 (50)	
	2015							CLSI					
												*Klebsiella* spp 30/35 (85.7)	
							Manual						
Wasihun^67^	Ethiopia	2014	Prospective cohort of febrile outpatients	Urban referral	All	NR	Manual	Disc diffusion	NR	Yes	NR	*E. coli* 9/16 (56.2)	
							Standard biochemical	CLSI					
	2015												
			Febrile, no antibiotics for 2 weeks										

CAI, CA infection; DRC, Democratic Republic of the Congo; EOS, early-onset sepsis; FSM, French Society of Microbiology; HAI, HA infection; HCAI, HCA infection; LOS, late-onset sepsis; NR, not reported.

### Data analysis

Prevalence is described as proportions of 3GC-R isolates, calculated from numbers of isolates of *E. coli, Klebsiella* spp., non-typhoidal *Salmonella* (NTS) or *Salmonella* Typhi tested against a 3GC and the number of resistant strains. Forest plots were generated, illustrating proportion estimates for each study with 95% CI calculated using the Wilson’s score method. The *I*^2^ statistic was calculated to quantify heterogeneity.

Our initial analysis plan aimed to calculate a pooled proportion of 3GC resistance for each pathogen, using random-effects meta-analysis with subanalysis by African region. However, high levels of heterogeneity amongst included studies precluded meaningful meta-analysis and we therefore present median prevalence of 3GC resistance for each pathogen, with corresponding IQR to provide an assessment of the wide range in resistance prevalence. Medians were calculated for sSA and for each African region as defined by the United Nations Statistics Division.^9^

Heterogeneity of proportion estimates was explored using predefined subgroup analysis by African region and a *post hoc* subgroup analysis by age group of study population. Visual inspection of resulting forest plots was carried out and a test for subgroup differences applied where visual inspection suggested a likely difference in subgroup proportion estimates and where more than two studies contributed to each subgroup. We additionally examined for trends in proportions estimates over time using visual inspection of forest plots, ordered by year of publication, and a linear meta-regression model. Analyses were conducted using R version 3.5.1 (R Foundation for Statistical Computing, Vienna, Austria).

### Risk of bias assessment

In terms of delineating a population estimate, we noted that the most likely risk of bias is patient selection. Additionally, the laboratory techniques and their implementation may differ in sensitivity and specificity and could also introduce bias. We modified the Critical Appraisal Skills Programme (CASP) checklist to design a risk-of-bias assessment to fit our research question, assessing risk of bias in patient recruitment and laboratory techniques used (Table S4). The assessment was performed by both R.L. and P.M. and any disagreements were resolved by consensus.

To explore for indirect evidence of publication bias, we examined 3GC resistance estimates against the number of isolates included in the study, as smaller studies may be subject to publication bias.

## Results

**Figure 1.**
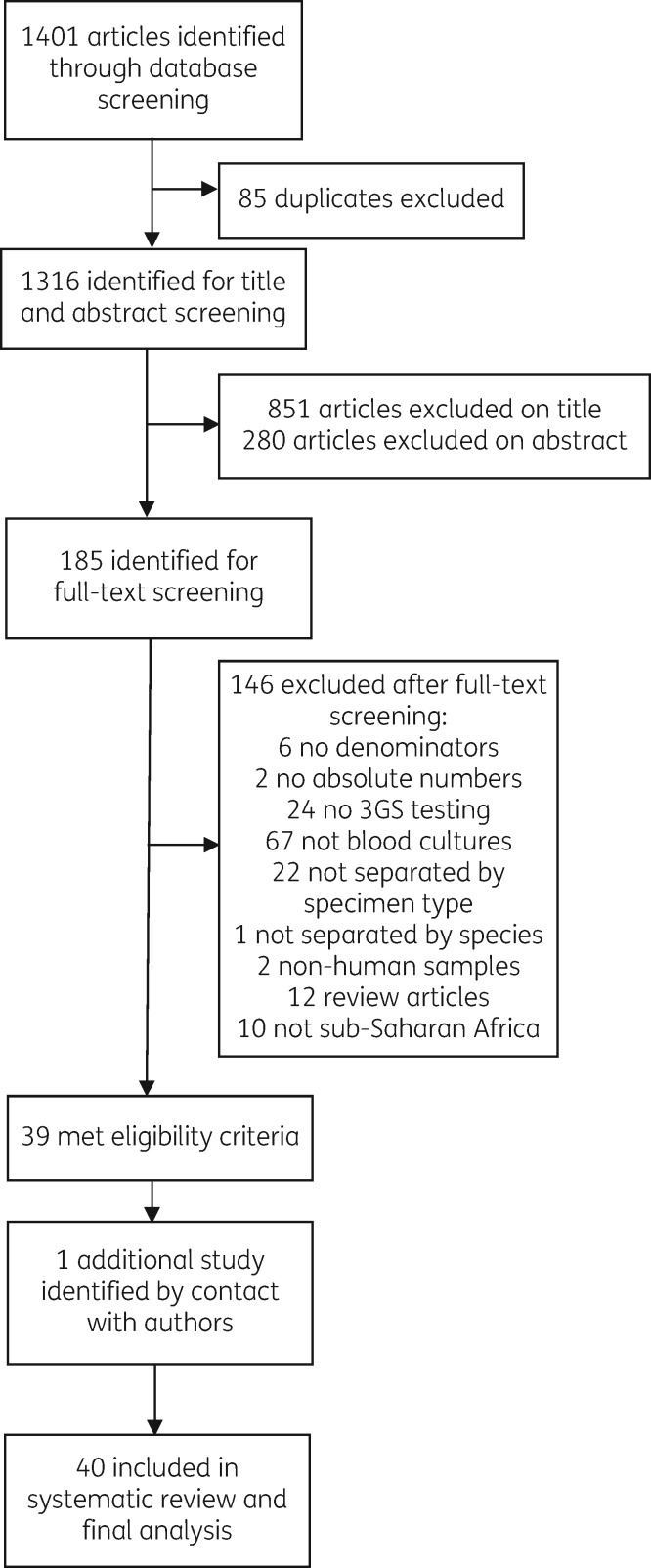
Study selection.

The online database search combined with reference review from key papers generated 1401 articles and, of these, 185 abstracts were selected for full-text review (Figure 1). Original data for one article were retrieved by direct communication with authors.^10^ Forty articles met the inclusion criteria and were included in the systematic review, which synthesizes 11 404 isolates. Of these, 20 articles reported proportions of 3GC resistance in *E. coli* and 28 in *Klebsiella* spp. Twelve studies reported proportions of 3GC resistance in NTS and four in *S*. Typhi.

**Figure 2.**
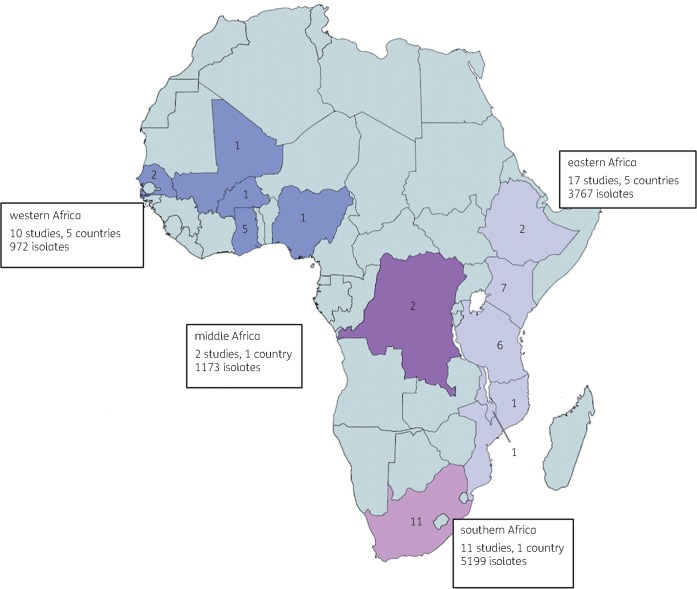
Geographical location of studies reporting proportions of 3GC resistance amongst *E. coli, Klebsiella* spp. and NTS. Numbers in country indicate the number of studies included in the review for each country. This figure appears in colour in the online version of *JAC* and in black and white in the print version of *JAC*.

Table 1 presents the characteristics of all included studies. Data were available from 12 countries across all four sSA regions (Figure 2), with the highest proportion of studies (11/40) from South Africa. All studies were observational. There were 30 studies that recruited cohorts of patients with confirmed or suspected BSI, 16 of which were prospective, 13 retrospective and 1 mixed. Four studies were cross-sectional reviews of isolates and three tested isolates collected as part of longitudinal multisite surveillance. There was one case–control study, designed to estimate mortality from 3GC-R BSI.^11^

**Figure 3.**
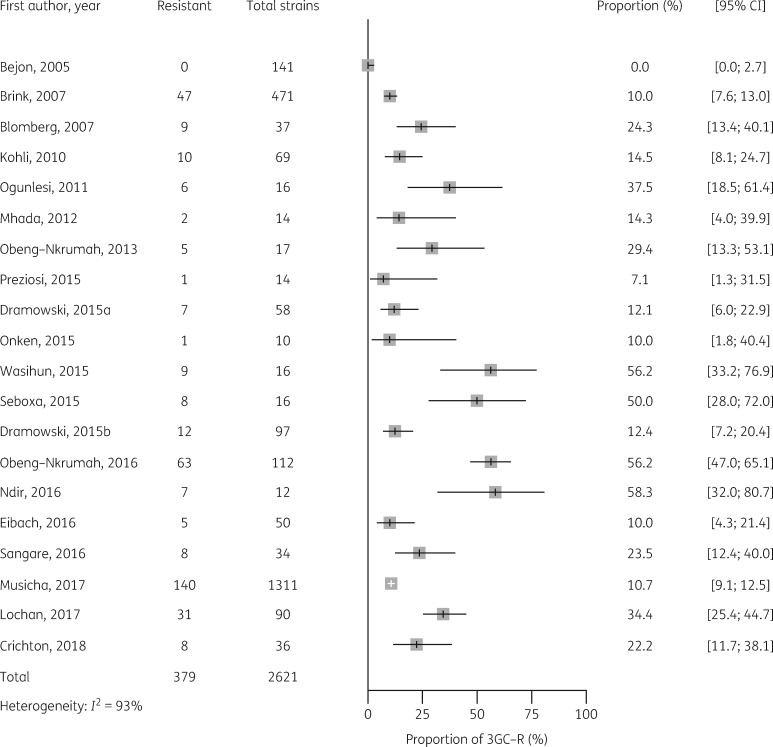
Proportion of 3GC resistance in 2621 *E. coli* BSI isolates from 20 studies.

**Figure 4.**
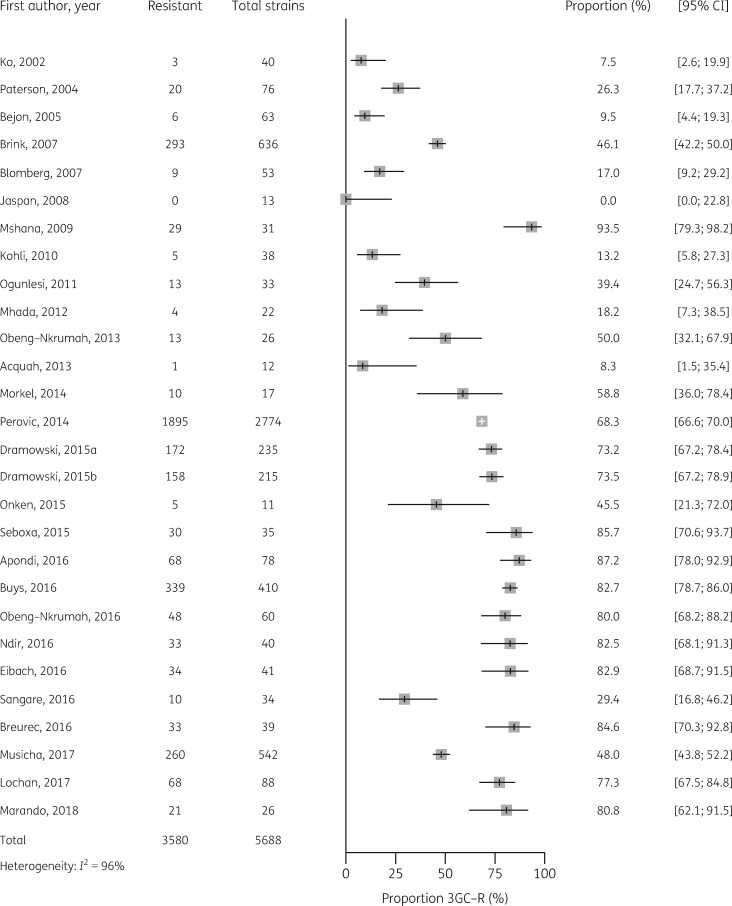
Proportion of 3GC resistance in 5688 *Klebsiella* spp. BSI isolates from 28 studies.

**Figure 5.**
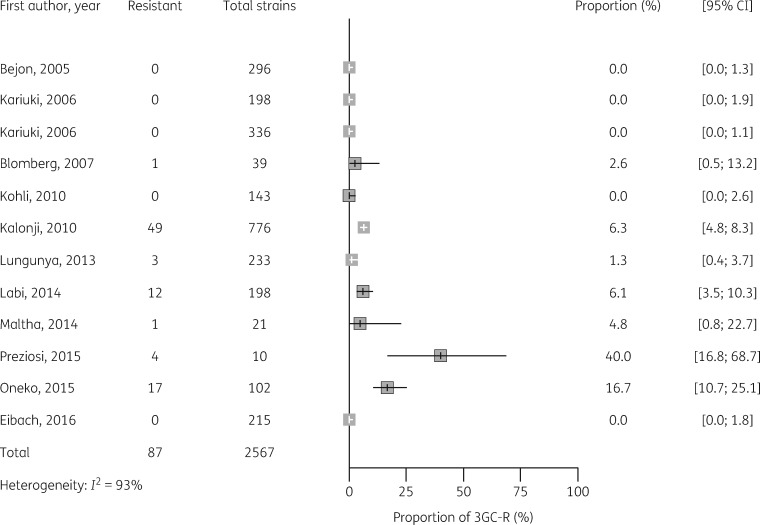
Proportion of 3GC resistance in 2567 NTS BSI isolates from 12 studies.

Median estimates of 3GC resistance in *E. coli, Klebsiella* spp. and salmonellae for sSA are shown in Table 2, together with median estimates by African region, and forest plots of individual studies are shown in Figures 3–5. The median point estimate of 3GC resistance in *E. coli* BSI from 20 studies was 18.4% (IQR 10.5 to 35.2) (Table 2). Heterogeneity was high (*I^2^* = 93%) (Figure 3) and not explained by prespecified subgroup analysis by African region (Figure S1). Median point estimates of 3GC resistance in *Klebsiella* BSI were higher across all regions than for *E. coli*, with an overall estimate of 54.4% (IQR 24.3 to 81.2) from 28 studies (Table 2, Figure 4). As with *E. coli*, heterogeneity was high (*I*^2^ =* * 96%) and not explained by differences in African region (Figure S1).

**Table 2.** Median prevalence of 3GC resistance in *E. coli*, *Klebsiella* spp. and NTS BSI, shown by African region

	Prevalence, % (IQR)				
Pathogen	overall 3GC resistance	eastern	middle	western	southern
*E. coli*	18.4 (10.5–35.2)	14.3 (10.0–24.3)	no data	33.5 (25.0–51.6)	12.4 (12.1–22.2)
	20 studies	9 studies		6 studies	5 studies
*Klebsiella* spp.	54.4 (24.3–81.2)	46.7 (17.3–84.5)	no data	58.3 (34.6–82.6)	63.6 (39.1–76.2)
	28 studies	10 studies		8 studies	10 studies
NTS	1.9 (0–6.1)	0 (0–9.6)	1.3, 6.3	4.8 (2.4–5.4)	no data
	12 studies	7 studies	2 studies	3 studies	

3GC resistance amongst NTS was low, at a median of 1.9% (IQR 0 to 6.1) in isolates from 12 studies (Figure 5). The highest proportions of 3GC resistance in NTS came from eastern Africa (Kenya and Mozambique) but subgroup analysis by African region did not explain interstudy variability (Figure S1). Four studies in this review carried out 3GC susceptibility testing on *S.* Typhi isolates.^12–15^ Of these, two studies from Kenya^12^ and Tanzania^14^ found 3GC resistance with prevalence of 6% (6/100) and 5.9% (1/17), respectively. These studies did not report confirmatory ESBL testing on cephalosporin-resistant *S*. Typhi strains.

The earliest published reports of 3GC resistance in Gram-negative BSI are from 2002.^16^ Graphical exploration of forest plots, ordered by year of publication (Figures 3–5), suggested a trend towards increased 3GC resistance over time for *Klebsiella*, NTS and *E. coli*. Meta-regression by year of publication supported a significant trend towards increased resistance over time for *Klebsiella* (*P*<0.01), NTS (*P*=0.02) and *E. coli* (*P*=0.02).

Studies reporting mortality estimates from 3GC-R BSI are shown in Table 3. Only one study, a paediatric case–control study in Senegal, was designed to determine attributable mortality from 3GC resistance as a primary outcome, finding that 3GC-R BSI remained the only significant independent risk factor for death in multivariable logistic regression, (OR=2.9, 95% CI 1.8–7.3, *P*=0.001) regardless of antibiotic treatment choice.^11^ Seven further studies^10^^,^^17–22^ provide mortality estimates for patients with 3GC-R BSI, but were not designed to estimate attributable mortality from these infections. These studies were a mixture of retrospective and prospective designs, variably providing ORs, RRs and case-fatality rates and incorporating different characteristics in multivariable models. It was therefore not possible to combine these into a single mortality estimate using meta-analysis. Where available, case-fatality rates from individual studies were high, ranging from 60% to 100%, with all but one study concluding 3GC-R BSI to be a predictor of fatal outcome in patients.

**Table 3.** Studies reporting mortality in patients with 3GC-R BSI

Study, publication year	Study type	Population	Country	Total patients in study	Pathogens	Case-fatality rate, 3GC-R 3GC-S *n* (%)	Adjusted mortality estimate from 3GC-R BSI (95% CI)	Author conclusions
Blomberg^17^	Prospective cohort	Paediatric; 0–7 years	Tanzania	1632	Mixture of Enterobacteriaceae	15/21 (71.0)	OR 12.87 (4.95–33.48)	Inappropriate antimicrobial therapy due to 3GC resistance predicts fatal outcome
						NR	Multivariable model adjusted for: age <1 month, sex, HIV status, malaria, other underlying disease, polymicrobial blood culture	
2007								
		Urban referral hospital						
		Children with suspected systemic infection based on IMCI						
Dramowski^10^	Retrospective cohort	Paediatric; 0–14 years	South Africa	864	Mixture of Enterobacteriaceae (mortality data available for *Klebsiella* spp.)	21/122 (17.2)	Not reported by AMR type	AMR not associated with BSI mortality
		Urban referral hospital						
2015								
						NR		
		Children with suspected sepsis or severe focal infection						
Onken^19^	Prospective cohort	All ages; no range reported	Zanzibar	469	Mixture of Enterobacteriaceae	3/5 (60.0)	Not reported	No significantly higher case-fatality rate in 3GC-R compared with susceptible infections, but small numbers
2015		Urban referral hospital				4/11 (36.0)		
		Patients with fever (≥38.3°C in adults, ≥38.5°C in children) or hypothermia (<36.0°C), tachypnoea >20/min, tachycardia >90/min or suspected systemic bacterial infection						
Seboxa^18^	Prospective cohort	Adults; 13–98 years	Ethiopia	232	Mixture of Enterobacteriaceae	11/11 (100)	RR 9.00 (1.42–57.12)	Inappropriate antimicrobial therapy due to 3GC-R infections predicts fatal outcome
2015		Urban referral hospital				1/9 (11.1)	No multivariable analysis	
		Patients with clinical suspicion of septicaemia and 2 of the 3 following criteria: axillary temperature ≥38.5°C or ≤36.5°C, pulse ≥90 beats/min and frequency of respiration ≥20/min						
Buys^21^	Retrospective cohort	Paediatric; IQR 2–16 months	South Africa	410	*Klebsiella* spp.	NR	OR 1.09 (0.55–2.16)	MDR *K. pneumoniae* BSI is associated with high mortality in children
		Urban referral hospital					Multivariable model adjusted for: age, gender, nutrition, HIV, ESBL, patient in PICU, patient needing to go to PICU, continuous IV infusion for >3 days before the BSI, *Klebsiella* BSI without source, chronic underlying medical condition excluding HIV, and skin erosions	
2016								
		Electronic list of *Klebsiella* bloodstream isolates from hospital database						
Eibach^20^	Prospective cohort	All ages; IQR 1–18 years	Ghana	7172	Mixture of Enterobacteriaceae	NR	Whole cohort: OR 3.0 (1.2–7.3) Neonates: OR 0.6 (0.1–3.7) No multivariable regression reported	3GC-R BSI is associated with higher mortality than non-3GC-R, but this is highly dependent on age
2016		Rural primary healthcare centre Patients with fever ≥38°C or history of fever within 24 h after admission or neonates with suspected neonatal sepsis						
								No mortality difference from 3GC-R infections in neonates and higher overall mortality

Ndir^11^	Case–control	Paediatric; 0–17 years	Senegal	173	Mixture of Enterobacteriaceae	NR (54.8)	OR 2.9 (1.8–7.3)	3GC-R BSI is associated with fatal outcome in HA-BSI
2016		Urban referral hospital				NR (15.4)	Multivariable model adjusted for: age <1 month, prematurity, underlying comorbidities, admission diagnoses, invasive procedures, inappropriate antibiotics	
		Cases—patients with an HA-BSI caused by Enterobacteriaceae						
		Controls—patients who did not experience an infection during the study period, randomly selected from the hospital database						
Marando^44^ 2018	Prospective cohort	Neonates; IQR 4–8 days	Tanzania	304	Mixture of Enterobacteriaceae	NR (34.4) NR	HR 2.4 (1.2–4.8), Cox regression	Neonates infected with 3GC-R BSI have significantly higher mortality than EBSL negative or non-bacteraemic patients
							OR 2.71 (1.22–6.03), multivariable model adjusted for age and sex	


3GC-S, 3GC susceptible; IMCI, integrated management of childhood infection.

Additional study population characteristics are shown in Table 1. There were 22 studies in paediatric populations, including 6 exclusively in neonates. Four studies recruited adults over 16 years of age, 13 recruited from all age groups and one study did not report age of participants from which blood cultures were obtained. Given that age categories were generally well reported and could explain differences between proportion estimates, we carried out *post hoc* stratified analysis by age group (Figure S2). Visual inspection of resulting forest plots suggested no difference in proportion estimates by age group for *E. coli* (Figure S2a), but potentially higher proportion estimates for 3GC-R *Klebsiella* in children than in adults (Figure S2b). A higher proportion estimate for 3GC resistance in NTS was seen in adults (Figure S2c) but there was only one study in this age group.

**Figure 6.**
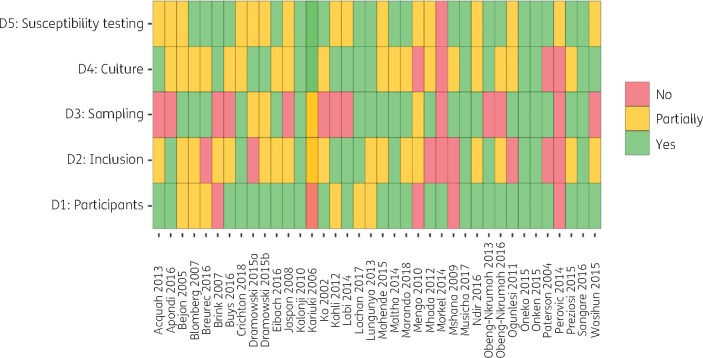
Results of risk-of-bias assessment. Domain 1: are the characteristics of participants adequately described? Domain 2: are the inclusion criteria explicit and appropriate? Domain 3: are the criteria for blood culture sampling explicit? Domain 4: are the blood culture methods precise and reported? Domain 5: are the AST methods precise and reported? This figure appears in colour in the online version of *JAC* and in black and white in the print version of *JAC.*

Results of the risk-of-bias assessment are shown in Figure 6. Bias in prevalence estimates was most likely introduced through selection of study participants. Many studies did not report criteria for blood culture sampling in the population recruited and many were conducted in special populations such as neonatal ICUs (NICUs). Most studies described blood culture methods well, but few reported external quality control (QC) in laboratory methods, resulting in a moderate risk of bias introduction across this domain for most studies.

As a measure of potential publication bias, plots of 3GC resistance estimates against study size, for *E. coli* and *Klebsiella* spp., are shown in Figure S2. For *E. coli* and *Klebsiella,* the larger studies tended to report lower resistance estimates (Figure S3), suggesting a potential for publication bias against studies reporting a smaller number of isolates.

Blood culture processing techniques varied. An automated system for blood culture incubation was used in 18 studies, whilst manual systems were used in 10. Three studies reported a mixture of manual and automated techniques and nine did not report which methods were used. AST methods varied, but most laboratories used disc diffusion (22/40). Four studies used VITEK 2, with the remainder using Etest, MicroScan or a mixture of techniques. Three studies did not report which AST methods were used. Most studies (30/40) used CLSI breakpoint guidelines, with the remainder using national or international guidelines as shown in Table 1. Twenty-two studies carried out ESBL confirmatory testing in 3GC-R isolates. Of these, 10 used double-disc synergy, with the remainder using broth dilution, PCR or a mixture of methods.

The classification of isolates by source, for example whether community-acquired (CA) or hospital-acquired (HA), or urban versus rural, is key to the interpretation of these data. Thirty studies tested BSIs from patients presenting to public referral or private hospitals in urban settings, with nine recruiting from rural district hospitals and one from a mixed urban/rural setting. HIV status of individuals who had blood culture sampling was recorded in only 11 studies and 1 study was exclusively a cohort of HIV-infected individuals. Six studies investigated the difference in blood culture pathogens and prevalence of resistance between CA and HA or healthcare-associated (HCA) infection. Of these, five found a higher prevalence of 3GC resistance in HA infections. Two studies were cohorts of patients with HA infection and one study included only patients with suspected CA BSI. Of the six neonatal studies, two differentiated early-onset from late-onset neonatal sepsis but did not report on differences in proportions of 3GC resistance between the two groups.

## Discussion

Our systematic review has synthesized over 11 000 blood culture isolates from patients in sSA, finding high levels of 3GC resistance amongst the key Enterobacteriaceae, *E. coli* and *Klebsiella* spp., and emerging resistance amongst salmonellae. Ceftriaxone is one of the most widely used broad-spectrum antibiotics in Africa, indicated in the empirical management of adult and paediatric patients at district-, regional- and tertiary-level care facilities.^23–25^ Limited access to carbapenems and aminoglycosides may make 3GC-R BSI untreatable in some settings.^8^ The striking lack of mortality data we describe in this review is therefore a major barrier to a comprehensive understanding of the burden of AMR in this setting.

We found a high median prevalence of 3GC resistance in *E. coli* BSI, greater than estimates from high-income countries, which are typically less than 10%.^26^ Interpreting the significance of proportion estimates in the absence of trend data is challenging and the latter will require long-term, high-quality surveillance. Some of the most comprehensive published trend data come from Malawi, where blood culture surveillance for 18 years has shown a recent, rapid rise in 3GC resistance amongst Enterobacteriaceae in adult^8^ and paediatric patients.^27^ Between 2003 and 2016, the proportion of 3GC-R *E. coli* rose from 0.7% to 30.3%, with similar trends in other non-*Salmonella* Enterobacteriaceae.^8^ The alarming trends described in Malawi highlight the urgent need for systematic AMR surveillance data from Africa that will inform both policy on access to antimicrobials and public health programmes aimed at reducing DRIs.

Resistance amongst *Klebsiella* spp., at 50.0%, was higher than for *E. coli*. *Klebsiella* spp. frequently acquire AMR genes and are a common cause of BSI in vulnerable populations, often causing localized outbreaks in settings such as NICUs and paediatric ICUs (PICUs).^28^ 3GC-R *Klebsiella* spp. are a particular challenge in neonatal infection as, in addition to the vulnerability of this age group to severe bacterial infection, many antimicrobials are either relatively contraindicated (e.g. chloramphenicol) or not locally available as IV agents (e.g. ciprofloxacin). In the single study from this review in which mortality from 3GC-R *Klebsiella* was recorded, all patients died; clearly, prospective studies investigating transmission dynamics of this nosocomial pathogen are required in order to support targeted interventions to reduce their development and spread.^21^

Although resistance to first-line antimicrobials, such as ampicillin, chloramphenicol and co-trimoxazole, is common among NTS in sSA,^29^ 3GC resistance has remained low, but may represent an emerging problem (Figure 5).^30^ Our review found sporadic cases of ceftriaxone resistance amongst *S*. Typhi from three countries, but these studies did not carry out confirmatory testing for the presence of ESBL genes. Although not captured by our inclusion criteria, ESBL-producing *S*. Typhi have been detected in sSA.^31^^,^^32^ In light of the recent outbreak of fluoroquinolone-resistant and ESBL-producing *S*. Typhi in Pakistan, resulting from the acquisition of ESBL-encoding plasmids by the H58 haplotype (genotype 4.3.1) known to be prevalent in Africa, this is concerning.^33^ Surveillance of *S*. Typhi non-susceptibility in Africa will be essential, as emergence of drug-resistant strains is associated with increase in transmissibility of typhoid and resurgence of disease.^34^

We found marked heterogeneity amongst 3GC resistance proportion estimates, which was not explained by differences in African region or age group of patients. Prevalence of resistance amongst key pathogens is likely to be influenced by a variety of clinical parameters including HIV status, healthcare attendance and prior antibiotic use, but these data were rarely reported and subgroup analysis by these factors was impossible. Detailed clinical and demographic parameters should be collected by studies that aim to understand the epidemiology of DRIs and the drivers of transmission of AMR pathogens.

We aimed to provide an estimate of the mortality burden from 3GC-R BSI, but this was prohibited by the scarcity of outcome data and heterogeneity of study designs. DRIs are associated with adverse patient outcomes in high-income settings, including high mortality and increased length of hospital stay.^35^^,^^36^ In Africa, where the prevalence of bacterial sepsis is high,^4^ late presentation to secondary care is common and the availability of alternative antimicrobials and advanced laboratory diagnostics is limited, the impact of AMR on patients is predictable, but currently unknown.

This review has a number of limitations. Heterogeneity is highly likely with reviews of this nature and the variety of populations described make a true general population estimate difficult. Potential sources of heterogeneity that we have not explored include the diversity of laboratory microbiological methods used, both for organism identification and for AST. Most studies did not report whether or how they engaged with external quality assurance programmes. We did not exclude these from the review, as they likely represent the vast majority of facilities in sSA, but this may be an important source of variation in estimates. Confirmatory testing for ESBL production using phenotypic or molecular methods is recommended for any organisms showing reduced susceptibility to an indicator 3GC, but such confirmatory methods were employed in just under half the studies included in this review. However, resistance to 3GCs on primary screening tests is sufficient evidence to infer 3GC resistance; therefore, again, we did not exclude these studies from the analysis. Our assessment of publication bias suggested a potential bias against publication of studies reporting on a small number of isolates. However, the differences in resistance estimates reported by studies of different sizes are much more likely explained by differences in the included populations, particularly since the majority of studies were not designed to estimate resistance, but reported estimates as part of blood culture surveillance or sepsis cohorts.

The limitations of available data we highlight in this review, together with the high level of unexplained interstudy heterogeneity, prompt the need for standardization of AMR research. In future, studies should be required to provide a clear account of the microbiological sampling criteria, study or surveillance sampling frame and laboratory methods used to generate resistance data. Studies should collect and report clinical metadata associated with the sample, including empirical antibiotic regimens, HIV status and the clinical setting, including level of the health system and intensity of care. There are increasing efforts in the AMR surveillance community to identify exactly which data are minimally acceptable and which data are ideal, to produce useful prevalence estimates that contribute to global repositories such as the WHO’s Global Antimicrobial Resistance Surveillance System (GLASS).^37^

We have documented proportions of 3GC-R BSI from a large number of bloodstream isolates across sSA, expanding on previous reviews that have focused on clinical syndromes,^38^ paediatric populations^39^ or limited African regions.^40^ Using inclusion criteria that captured surveillance studies in addition to clinical cohorts, we have, to our knowledge, captured the largest AMR dataset available from sSA and therefore provide the most comprehensive summary of 3GC-R BSI from the continent. In doing so, we demonstrate the lack of available clinical data and show that the burden of DRIs on patients in Africa remains unknown. Low-income countries have multiple, competing priorities for limited healthcare resources and budgets, therefore clinicians, researchers and policymakers will need to demonstrate that AMR is a priority for patients in these settings. This information does not currently exist and AMR prevalence studies from sSA, however comprehensive, will need to be accompanied by robust morbidity, mortality and economic outcome data, to allow for a true understanding of the burden of AMR on patients and health systems.

## Funding

This work was supported by the Wellcome Trust (Clinical PhD Fellowship to R.L., University of Liverpool block award grant number 203919/Z/16/Z).

## Transparency declarations

None to declare.

## Supplementary Material

dkz464_Supplementary_Data
