# Supplementary material for: Prevalence and outcome of bloodstream infections due to third-generation cephalosporin-resistant Enterobacteriaceae in sub-Saharan Africa: a systematic review
Source: J Antimicrob Chemother. 2019 Nov 19;75(3):492–507. doi: 10.1093/jac/dkz464 (PMC7021093; doi:10.1093/jac/dkz464)
Supplement: dkz464_Supplementary_Data [file dkz464_supplementary_data.docx]

**Supplementary data**

**Table S1:** Screening strategy as per study protocol

We assessed eligibility by reviewing title and abstract, and subsequently by reviewing the full text.

| **Inclusion criteria** |
| --- |
| Isolates are tested for susceptibility to a third-generation cephalosporin (3GC) (ceftriaxone, cefpodoxime, cefotaxime, ceftazidime, or cefixime)  Isolates tested must be either: *E coli,* *Klebsiella* spp., or *Salmonella* spp.  The samples tested must be blood cultures |
| **Exclusion criteria** |
| - The publication is a review article, a case report, a commentary, or an editorial.  - 3GC-R is not clearly reported as a proportion (number of samples resistant over total isolates tested against the antibiotic), or this information cannot be obtained from the authors |

| **Table S2. PRISMA Checklist** | | | | |  | |
| --- | --- | --- | --- | --- | --- | --- |
| **Section/Topic** | **Checklist item** | | | | **Reported on page #** | |
| **TITLE** | | | | |  | |
| Title | 1 | | Identify the report as a systematic review, meta-analysis, or both. | | 1 | |
| **ABSTRACT** | | | | |  | |
| Structured summary | 2 | | Provide a structured summary including, as applicable: background; objectives; data sources; study eligibility criteria, participants, and interventions; study appraisal and synthesis methods; results; limitations; conclusions and implications of key findings; systematic review registration number. | | 2 | |
| **INTRODUCTION** | | | | |  | |
| Rationale | 3 | | Describe the rationale for the review in the context of what is already known. | | 3-4 | |
| Objectives | 4 | | Provide an explicit statement of questions being addressed with reference to participants, interventions, comparisons, outcomes, and study design (PICOS). | | 4 | |
| **METHODS** | | | | |  | |
| Protocol and registration | 5 | | Indicate if a review protocol exists, if and where it can be accessed (e.g., Web address), and, if available, provide registration information including registration number. | | 4  Included in supplementary material | |
| Eligibility criteria | 6 | | Specify study characteristics (e.g., PICOS, length of follow-up) and report characteristics (e.g., years considered, language, publication status) used as criteria for eligibility, giving rationale. | | 4 | |
| Information sources | 7 | | Describe all information sources (e.g., databases with dates of coverage, contact with study authors to identify additional studies) in the search and date last searched. | | 4 | |
| Search | 8 | | Present full electronic search strategy for at least one database, including any limits used, such that it could be repeated. | | Supplementary material | |
| Study selection | 9 | | State the process for selecting studies (i.e., screening, eligibility, included in systematic review, and, if applicable, included in the meta-analysis). | | 4 | |
| Data collection process | 10 | | Describe method of data extraction from reports (e.g., piloted forms, independently, in duplicate) and any processes for obtaining and confirming data from investigators. | | 5 | |
| Data items | 11 | | List and define all variables for which data were sought (e.g., PICOS, funding sources) and any assumptions and simplifications made. | | 5 | |
| Risk of bias in individual studies | 12 | | Describe methods used for assessing risk of bias of individual studies (including specification of whether this was done at the study or outcome level), and how this information is to be used in any data synthesis. | | 6 | |
| Summary measures | 13 | | State the principal summary measures (e.g., risk ratio, difference in means). | | 5 | |
| Synthesis of results | 14 | | Describe the methods of handling data and combining results of studies, if done, including measures of consistency (e.g., I^2^) for each meta-analysis. | | 6 | |
| Risk of bias across studies | 15 | | Specify any assessment of risk of bias that may affect the cumulative evidence (e.g., publication bias, selective reporting within studies). | | Not done | |
| Additional analyses | 16 | | Describe methods of additional analyses (e.g., sensitivity or subgroup analyses, meta-regression), if done, indicating which were pre-specified. | | 6 | |
| **RESULTS** | | | | |  | |
| Study selection | 17 | | Give numbers of studies screened, assessed for eligibility, and included in the review, with reasons for exclusions at each stage, ideally with a flow diagram. | | 6 and Figure 1 | |
| Study characteristics | 18 | | For each study, present characteristics for which data were extracted (e.g., study size, PICOS, follow-up period) and provide the citations. | | Table 1 | |
| Risk of bias within studies | 19 | | Present data on risk of bias of each study and, if available, any outcome level assessment (see item 12). | | 8-9  Figure 6 | |
| Results of individual studies | 20 | | For all outcomes considered (benefits or harms), present, for each study: (a) simple summary data for each intervention group (b) effect estimates and confidence intervals, ideally with a forest plot. | | Figures 3-5 | |
| Synthesis of results | 21 | | Present results of each meta-analysis done, including confidence intervals and measures of consistency. | | NA | |
| Risk of bias across studies | 22 | | Present results of any assessment of risk of bias across studies (see Item 15). | | NA | |
| Additional analysis | 23 | | Give results of additional analyses, if done (e.g., sensitivity or subgroup analyses, meta-regression [see Item 16]). | | Supplementary material | |
| **DISCUSSION** | | | | |  | |
| Summary of evidence | 24 | | Summarize the main findings including the strength of evidence for each main outcome; consider their relevance to key groups (e.g., healthcare providers, users, and policy makers). | | 10-12 | |
| Limitations | 25 | | Discuss limitations at study and outcome level (e.g., risk of bias), and at review-level (e.g., incomplete retrieval of identified research, reporting bias). | | 10-12 | |
| Conclusions | 26 | | Provide a general interpretation of the results in the context of other evidence, and implications for future research. | | 10-12 | |
| **FUNDING** | | | | |  | |
| Funding | 27 | | Describe sources of funding for the systematic review and other support (e.g., supply of data); role of funders for the systematic review. | | 14 | |
| Risk of bias across studies | | 15 | | Specify any assessment of risk of bias that may affect the cumulative evidence (e.g., publication bias, selective reporting within studies). | | Not done |
| Additional analyses | | 16 | | Describe methods of additional analyses (e.g., sensitivity or subgroup analyses, meta-regression), if done, indicating which were pre-specified. | | 6 |
| **RESULTS** | | | | | |  |
| Study selection | | 17 | | Give numbers of studies screened, assessed for eligibility, and included in the review, with reasons for exclusions at each stage, ideally with a flow diagram. | | 6 and Figure 1 |
| Study characteristics | | 18 | | For each study, present characteristics for which data were extracted (e.g., study size, PICOS, follow-up period) and provide the citations. | | Table 1 |
| Risk of bias within studies | | 19 | | Present data on risk of bias of each study and, if available, any outcome level assessment (see item 12). | | 8-9  Figure 6 |
| Results of individual studies | | 20 | | For all outcomes considered (benefits or harms), present, for each study: (a) simple summary data for each intervention group (b) effect estimates and confidence intervals, ideally with a forest plot. | | Figures 3-5 |
| Synthesis of results | | 21 | | Present results of each meta-analysis done, including confidence intervals and measures of consistency. | | NA |
| Risk of bias across studies | | 22 | | Present results of any assessment of risk of bias across studies (see Item 15). | | NA |
| Additional analysis | | 23 | | Give results of additional analyses, if done (e.g., sensitivity or subgroup analyses, meta-regression [see Item 16]). | | Supplementary material |
| **DISCUSSION** | | | | | |  |
| Summary of evidence | | 24 | | Summarize the main findings including the strength of evidence for each main outcome; consider their relevance to key groups (e.g., healthcare providers, users, and policy makers). | | 10-12 |
| Limitations | | 25 | | Discuss limitations at study and outcome level (e.g., risk of bias), and at review-level (e.g., incomplete retrieval of identified research, reporting bias). | | 10-12 |
| Conclusions | | 26 | | Provide a general interpretation of the results in the context of other evidence, and implications for future research. | | 10-12 |
| **FUNDING** | | | | | |  |
| Funding | | 27 | | Describe sources of funding for the systematic review and other support (e.g., supply of data); role of funders for the systematic review. | | 14 |

# Table S3: Search strategy

| MeSH search terms | “Drug resistance” OR “Antimicrobial resistance” OR  “Bacterial resistance” OR “Drug Resistance, Bacterial", OR “Microbial  Sensitivity Tests OR “Drug Resistance, Microbial”  **AND** |
| --- | --- |
| text word search | text word search: “antibiotic resistan*” OR “antibacterial  resistan*” OR “antimicrobial resistan*” OR “antimicrobial drug  resistan*” OR “antibiotic drug resistan*” OR “antibacterial drug  resistan*” OR “bacteraemia” OR “bacteremia” OR “bloodstream inf*”  **AND** |
| title and abstract | “angola” OR “benin” OR “botswana” OR burkina  faso” OR “burundi” OR “cameroon” OR “cape verde” OR “central  african republic” OR “chad” OR “ivory coast” OR “cote d ivoire” OR  “congo” OR “comoros” OR “djibouti” OR “equatorial guinea” OR  “eritrea” OR “ethiopia” OR “gabon” OR “gambia” OR “ghana” OR  “guinea” OR “guinea bissau” OR “kenya” OR “leshoto” OR liberia” OR  “madagascar” OR”malawi” OR “Mali” OR “mauritania” OR  “mozambique” OR “namibia” OR “niger” OR “nigeria” OR “rhodesia”  OR “rwanda” OR “sao tome” OR “sengal” OR “seychelles” OR “sierra  leone” OR “somalia” OR “south africa” OR “sudan” “swaziland” OR  “tanzania” OR “togo” OR “uganda” OR “zambia” OR “zimbabwe” OR  “africa” |

# Table S4: Risk of bias tool

Adapted from CASP checklists, and: Development of a quality appraisal tool for case series using a modified Delphi technique (Institute of Health Economics) 2012.^1,2^

| **Domain 1: Study population/participant recruitment**  **1.** Are the characteristics of the participants included in the study adequately described?  **Yes**: The authors should report the total number, age, and gender distribution of the participants who had blood cultures taken.    **Partially reported**: The criteria above are incompletely reported  **No**: None of the relevant characteristics of the participants is reported. |
| --- |
| **Domain 2. Are the eligibility criteria (inclusion and exclusion criteria) to enter the study explicit and appropriate?**  **Yes**: The eligibility criteria are clearly stated and replicable. A statement on age of eligibility is given. Blood cultures are taken from community and not hospital and not in a special population such as neonatal intensive care unit (NICU) or intensive care unit (ICU).  **Partially reported**: The criteria above are incompletely reported  **No**: The eligibility criteria are not clearly stated or are inappropriate. |
| **Domain 3. Was the method of recruitment adequately described and appropriate?**  **Yes**: There is a clear statement of the criteria for blood culture sampling of participants (e.g. patients with suspected systemic infection, or fever criteria) and the criteria are appropriate.  **Partially reported**: The criteria used for blood culture sampling are is not clearly stated or no information is provided. The criteria are vague.  **No**: The criteria for blood culture sampling are not given or were inappropriate – for example not done on patients with suspected infection, or dependent on access to resources or availability. |
| **Domain 4. Were the blood culture results precise and reported?**  **Yes**: There is a detailed description of the method of blood culture processing (e.g. Bactec). There is a detailed description of the method used for organism identification (e.g. API*). There is a statement confirming the use of external laboratory quality control.  **Partially**: The method of blood culture processing is vague or partially reported (eg.Bactec machine used for incubation, but method of identification not reported). External quality control is not reported.  **No**: The method of blood culture processing is not reported or quality control was not done  *Analytical profile index |
| **Domain 5. Were the antibiotic sensitivity testing (AST) methods precise?**  **Yes**: There is a detailed description of the method of AST and this follows recognized national or international guidelines. Confirmatory 3GS resistance testing was done  **Partially:** Method of AST testing vague or partially reported.  **No:** Method of AST testing not reported or do not follow recognized guidelines. |

**Figure S1 (a-c): Subgroup analysis of included studies by Africa region**

| **  (a) *E. coli:* Test for subgroup differences (random effects model) $Q$=3.09, d.f = 2, p=0.21 |  |
| --- | --- |
| ****  (b*) Klebsiella spp.* Test for subgroup differences (random effects model) $Q$=0.46, d.f = 2, p=0.79 |  |

| ****  (c) Non-typhoidal *Salmonella*: Test for subgroup differences (random effects model) $Q$=0.10, d.f = 2, p=0.95 |  |
| --- | --- |

**Figure S2 (a-c): Subgroup analysis of included studies by age-group of participant**

(a*) E.coli*

|  |  |
| --- | --- |
| **  (b*) Klebsiella spp.* |  |
| ****  (c*)* non-typhoidal *Salmonella* |  |

1. E.coli **B.** Klebsiella spp. **C**. NTS

|  |  |  |
| --- | --- | --- |

**Figure S3.** Plots of 3GC-R estimates against number of isolates included in each study, for (A) Ê.coli, (B) Klebsiella and (C) NTS, intended as an indicator of publication bias.

1. Development of a quality appraisal tool for case series studies using a modified Delphi technique. Alberta, Canada: Institute of Health Economics. *Economics IoH* 2012.

2. Stockdale AJ, Saunders MJ, Boyd MA, et al. Effectiveness of Protease Inhibitor/Nucleos(t)ide Reverse Transcriptase Inhibitor-Based Second-line Antiretroviral Therapy for the Treatment of Human Immunodeficiency Virus Type 1 Infection in Sub-Saharan Africa: A Systematic Review and Meta-analysis. *Clin Infect Dis* 2018; **66**(12): 1846-57.
